# The Potential Therapeutic Applications of Natural Products in the Oxidative Stress-Related MVA Pathway: Focus on HMGCR

**DOI:** 10.3390/antiox14081001

**Published:** 2025-08-16

**Authors:** Yu-Ning Teng

**Affiliations:** 1 Department of Pharmacy, College of Pharmacy, China Medical University, 100, Sec. 1, Jingmao Rd., Beitun Dist., Taichung City 406040, Taiwan; ynteng@mail.cmu.edu.tw or eunicegh520@gmail.com; Tel.: +886-4-22053366 (ext. 5158); 2 Department of Pharmacy, E-Da Cancer Hospital, 21 Yida Road, Kaohsiung 82445, Taiwan

**Keywords:** HMGCR, natural products, dyslipidemia, cholesterol, statins, cancer, oxidative stress

## Abstract

This review explores the therapeutic promise of natural compounds in modulating 3-hydroxy-3-methylglutaryl-coenzyme A reductase (HMGCR), a key enzyme in cholesterol synthesis. HMGCR dysregulation is implicated in dyslipidemia, cardiovascular disease, and cancer, conditions linked to oxidative stress. While statins inhibit HMGCR, their side effects necessitate exploring alternatives. The review highlights various natural compounds—flavonoids, phenolic acids, stilbenes, and herbal formulations—with HMGCR-modulating and antioxidant capabilities. In vitro and in vivo studies suggest these compounds offer a promising avenue for treating HMGCR-related conditions. Synergistic effects are observed when combining natural products with statins, hinting at combination therapies that could lower statin dosages and reduce adverse effects. Natural HMGCR modulators hold therapeutic promise but face hurdles like limited in vivo data, regulatory issues, variability in composition, potential drug interactions, and safety concerns. Future research must prioritize comprehensive mechanistic studies, standardized preparations, and well-designed clinical trials. Overcoming these challenges through rigorous science is essential for integrating natural HMGCR modulators into clinical practice and improving patient outcomes in a safe and effective manner. Specifically, clinical trials should consider combination therapies and comparison with standard treatments like statins. More research is also needed on optimal dosages and treatment regimens.

## 1. Introduction

Oxidative stress, resulting from an overproduction of reactive oxygen species (ROS) exceeding antioxidant defenses, emerges as a central mechanism linking dyslipidemia and cancer. Oxidative stress, which can arise from lipid alterations, activates inflammatory pathways and transcription factors involved in cell transformation, tumor survival, and angiogenesis. Specifically, alterations in serum lipid levels, such as increased total cholesterol, low-density lipoprotein cholesterol (LDL-C), and triglycerides (TGs), have been implicated in carcinogenesis [1]. Increased total cholesterol increases cellular proliferation and angiogenesis while inhibiting apoptosis. Increased LDL-C induces inflammation and heightened susceptibility to oxidative damage. Conversely, high-density lipoprotein cholesterol (HDL-C) exhibits antioxidative, anti-inflammatory, and antiproliferative properties [2]. Furthermore, elevated levels of TG can induce a chronic inflammatory state through oxidative stress, promoting cancer cell proliferation and progression. In contrast, statins influence cholesterol synthesis, which is crucial to cell proliferation and growth, demonstrating that lipid components could be prognostic for cancer and that management of lipid levels through lifestyle changes and medical therapy could be beneficial for cancer prevention and treatment [3].

**Figure 1:**
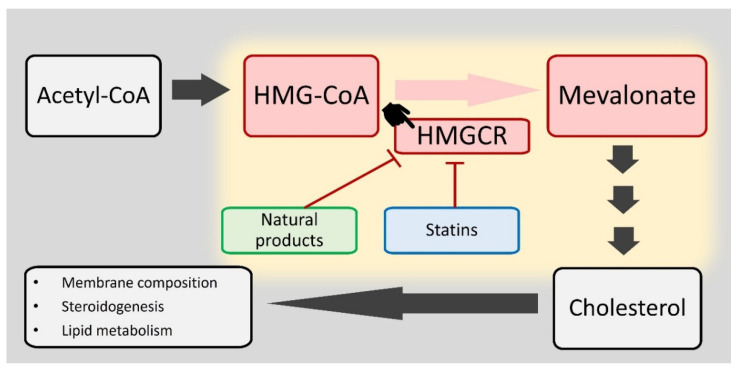
HMGCR catalyzes the rate-limiting step in the mevalonate pathway, a critical route for the synthesis of cholesterol. Cholesterol, in turn, is essential for maintaining membrane composition, steroid hormone biosynthesis (steroidogenesis), and lipid metabolism. HMGCR facilitates the conversion of HMG-CoA to mevalonate, a key intermediate in cholesterol synthesis. Consequently, HMGCR is a pharmacological target, with statins and various natural products known to inhibit its activity.

The mevalonate pathway (MVA), crucial for isoprenoid biosynthesis including cholesterol, commences with the condensation of acetyl-CoA to form 3-hydroxy-3-methylglutaryl-CoA (HMG-CoA). The rate-limiting step in this pathway is the conversion of HMG-CoA to mevalonate, a reaction catalyzed by HMG-CoA reductase (HMGCR) (Figure 1). This step commits the pathway to the synthesis of cholesterol and other isoprenoids, which are vital for maintaining cellular homeostasis through their roles in membrane integrity, steroid hormone production, and bile acid synthesis. Due to its central role, the mevalonate pathway is a key target for therapeutic intervention; statins, for example, inhibit HMGCR activity and are used to manage hypercholesterolemia [4,5].

HMGCR, the rate-limiting enzyme in the mevalonate pathway, directly regulates cholesterol biosynthesis by catalyzing the conversion of HMG-CoA to mevalonate, thereby determining the flux and efficiency of the pathway. The regulation of HMGCR is essential for maintaining cholesterol homeostasis, which influences cellular membrane composition, steroidogenesis, and overall lipid metabolism. Sterols exert feedback inhibition on HMGCR, modulating cholesterol synthesis based on cellular requirements [4]. Statins, which target HMGCR, are effective in lowering cholesterol levels, underscoring the enzyme’s therapeutic importance in managing cardiovascular disease [6].

Given its central role in cholesterol biosynthesis, HMGCR is a significant therapeutic target for dyslipidemia and related cardiovascular diseases. Statin-mediated inhibition of HMGCR effectively reduces low-density lipoprotein (LDL) cholesterol levels, thus reducing cardiovascular risk in patients with dyslipidemia [5,7,8]. In addition to LDL reduction, statins exhibit pleiotropic effects, such as improving endothelial function, reducing oxidative stress, and exerting anti-inflammatory actions [9], which further highlight the therapeutic value of targeting HMGCR in the prevention of cardiovascular morbidity and mortality.

Although statins are a cornerstone in the management of dyslipidemia, limitations exist, including adverse effects such as myopathy, liver enzyme abnormalities, and gastrointestinal issues, as well as suboptimal low-density lipoprotein cholesterol (LDL-C) reduction in certain patients [10,11]. This necessitates the exploration of alternative therapeutic strategies. Lovastatin, initially isolated from *Aspergillus terreus* cultures, exhibits a more potent inhibitory effect compared to mevastatin, as demonstrated by Alberts et al. [12,13]. This compound, originally identified as mevinolinic acid, represents the acid form of lovastatin, the active ingredient in Mevacor^®^, and serves as a precursor in the synthesis of simvastatin. Natural products, including those derived from red yeast rice, berberine, and omega-3 fatty acids, offer potential lipid-lowering benefits with a possibility of fewer side effects, and may be used to complement statin therapy [14,15,16]. Natural products are structurally diverse compounds produced by living organisms, including plants, microorganisms, and animals, often serving as secondary metabolites not directly involved in primary metabolic processes. These compounds encompass a wide array of chemical classes, such as alkaloids, terpenoids, flavonoids, and polyketides. Within the broader category of natural products, bioactive compounds are defined as specific constituents that exhibit demonstrable biological effects, such as pharmacological activity, in in vitro or in vivo systems. Identifying and isolating these bioactive compounds from complex mixtures of natural products is a crucial step in drug discovery and development [17].

This review provides a comprehensive assessment of the existing literature concerning natural products that modulate HMGCR activity for the management of dyslipidemia and related diseases. Considering the limitations associated with statin therapy, alternative therapeutic approaches utilizing natural compounds are of critical importance. This review systematically analyzes the mechanisms by which phytosterols, flavonoids, and other bioactive compounds influence HMGCR activity and cholesterol metabolism, summarizing their potential therapeutic applications and highlighting specific details and directions for future research endeavors.

## 2. Methods

**Figure 2:**
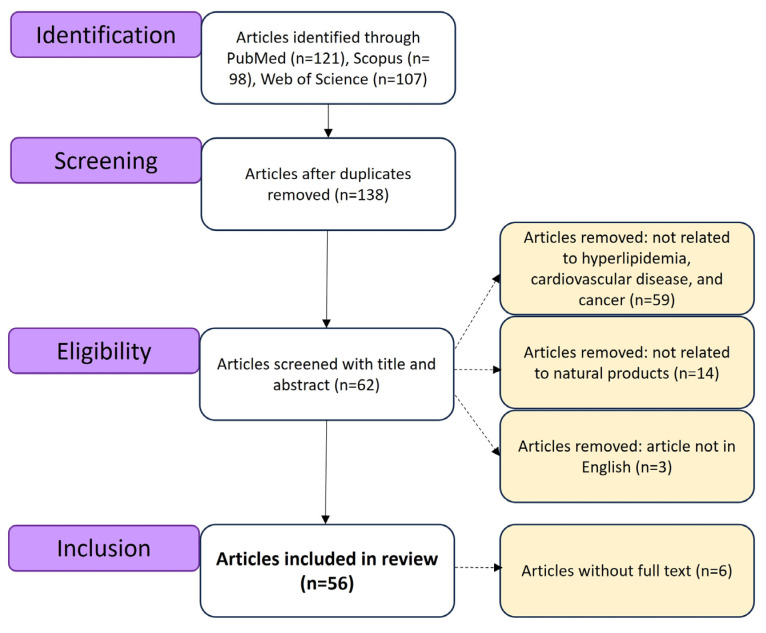
The flow chart of article selection.

A thorough literature search was conducted across the PubMed, Scopus, and Web of Science databases using the following keywords: “natural products”, “3-Hydroxy-3-Methylglutaryl-CoA Reductase”, “clinical disease”, “bioactive compounds”, “natural resources”, “mevalonate pathway”, and “MVA pathway”. The search covered publications from the year 2000 to 2025 and was carried out between 27 February 2025, and 26 March 2025. Studies were selected based on the following criteria: (1) published on or before 26 March 2025; (2) written in English; (3) accessible in electronic format with a digital object identifier (DOI); and (4) relevant to the disease-related topics addressed in this review. The article selection process is illustrated in Figure 2.

## 3. Therapeutic Applications

**Figure 3:**
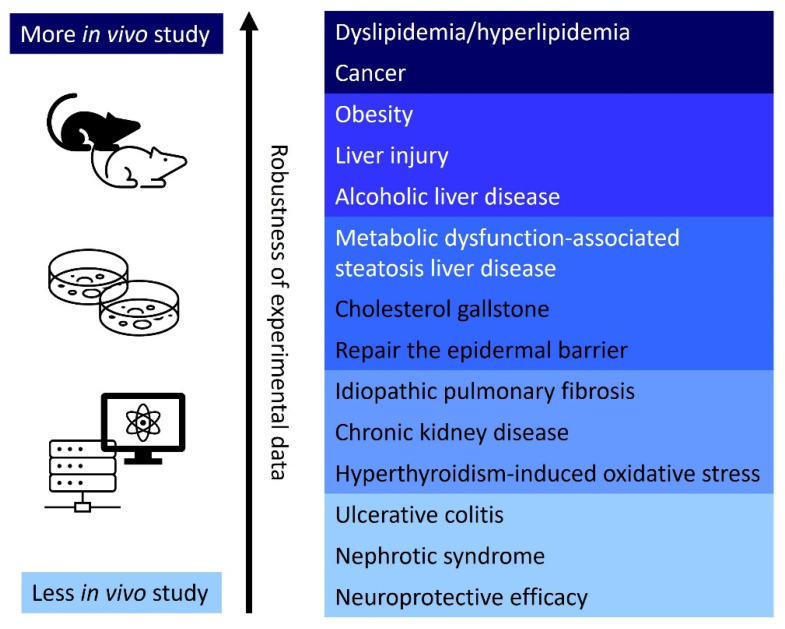
Experimental data supporting the use of natural products for clinical diseases related to HMGCR regulation. A review of the current literature indicates a preponderance of in vivo studies focused on the application of these natural products in the treatment of dyslipidemia/hyperlipidemia and cancer.

Based on current research, the regulation of HMGCR by natural products has implications for a variety of diseases. However, robust in vivo evidence, such as well-established animal models, currently supports the role of natural products in dyslipidemia, cancer, and obesity. Other potential applications have primarily been investigated through in vitro and in silico analyses, including network pharmacology and molecular docking studies (Figure 3). This review focuses on key findings related to the most significantly influenced areas, including dyslipidemia/hyperlipidemia, cardiovascular diseases, and cancer.

### 3.1. Dyslipidemia/Hyperlipidemia (Also Summarized in Table 1)

#### 3.1.1. Modulation of HMGCR Expression by Flavonoids and Phenolic Compounds

A substantial body of evidence highlights the ability of various flavonoids and phenolic compounds derived from plant sources to modulate HMGCR expression, thus influencing cholesterol synthesis. For example, studies on extracts from *Coreopsis tinctoria* Nutt., specifically focusing on luteolin, marein, naringenin (NGN), and chlorogenic acid (CQA), demonstrated their capacity to downregulate HMGCR expression in HepG2 cells exposed to oleic acid (OA), a model system for hyperlipidemia. This downregulation was associated with improved lipid profiles and reduced oxidative stress, suggesting the potential of these flavonoids in the management of hyperlipidemia [18]. Similarly, forest onion extract (FOE) has demonstrated the ability to downregulate HMGCR protein expression in vitro, contributing to the available treatment options for hyperlipidemia [19].

#### 3.1.2. Impact of Traditional Chinese Medicine (TCM) and Herbal Formulations on HMGCR

Traditional Chinese Medicine formulations provide a holistic approach to the management of hyperlipidemia, often attributed to the synergistic action of multiple bioactive components. Sanhua Jiangzhi Granules (SJG, a TCM formulation known for its lipid metabolism-regulating properties, has demonstrated the ability to downregulate HMGCR protein expression in the livers of rats, resulting in decreased body weight, improved blood lipid levels, and amelioration of liver pathology [20]. This effect on HMGCR appears to be mediated through the PPAR (peroxisome proliferator-activated receptors) signaling pathway, underscoring the intricate interactions of these compounds with cellular regulatory mechanisms. By mediating fatty acid transport proteins (FATPs) and fatty-acid translocase (FAT/CD36), SJG activates the PPAR pathway, influencing lipid metabolism in the liver and skeletal muscle via fatty-acid-binding proteins (FABPs). Consequently, nuclear PPARα stimulation leads to a decrease in SCD, HMGCR, and FABP1 expression, coupled with an increase in CYP7A1 and CPT-1 expression. In a similar vein, a water extract of *Ulmus macrocarpa* Hance (UME) downregulates hepatic HMGCR, suggesting its potential as a therapeutic agent for hyperlipidemia [21].

#### 3.1.3. Role of Specific Natural Products in HMGCR Regulation

Numerous studies have investigated the effects of individual natural products on HMGCR expression. For example, Radix *Angelica dahuricae* (RAD) extract has been shown to increase HMGCR expression and improve lipid profiles [22]. Quercetin, a phytochemical derived from buckwheat, demonstrates a more pronounced effect at higher concentrations, reducing cholesterol levels and downregulating HMGCR expression. This effect is dose-dependent and exhibits synergy when combined with simvastatin [23]. Further research has demonstrated that sinapic acid significantly attenuates the impact of a high-fat diet on HMGCR and other genes involved in lipid metabolism, suggesting a potential role in the management of dyslipidemia [24]. Furthermore, *Berberis aristata* extract has been shown to reduce proprotein convertase subtilisin/kexin type 9 (PCSK9), a key regulator of LDL receptor (LDLR) expression [25]. These examples underscore the diverse array of natural compounds capable of modulating HMGCR.

#### 3.1.4. Influence of Food Processing and Fermentation on HMGCR-Related Effects

Processing techniques applied to foods and herbs can substantially alter their bioactive components and, consequently, their effects on lipid metabolism. For example, heat-processing of *Gynostemma pentaphyllum* enhances its lipid-lowering effects, with network pharmacology analyses identifying HMGCR as a key target [26]. Furthermore, fermenting *Rhus verniciflua* Stokes with *Saccharomyces carlsbergensis* increases its lipid-lowering potential, as evidenced by a significant suppression of lipid accumulation and reduced HMGCR expression in HepG2 cells [27]. These findings highlight the importance of considering processing methods when assessing the efficacy of natural products.

#### 3.1.5. Modulation of HMGCR Through the AMPK Pathway

The AMP-activated protein kinase (AMPK) pathway is a critical regulator of cellular energy balance and lipid metabolism. A number of natural products, including extracts from forest onion (*Eleutherine bulbosa* Merr.), have been shown to activate AMPK, which, in turn, leads to the downregulation of HMGCR and a subsequent reduction in cholesterol synthesis [19]. In 3T3-L1 mouse cells, FOE dose-dependently inhibits MAPK8, PPARG, HMGCR, CPT-1, and GLP-1 expression. AMPK, an upstream regulator, phosphorylates and inhibits HMGCR, connecting energy status to lipid metabolism by reducing cholesterol synthesis. Given their opposing roles in lipid metabolism, AMPK activation often reduces PPARG activity, which is involved in fat cell differentiation and glucose metabolism. This AMPK-mediated mechanism appears to be a common pathway underlying the hypolipidemic effects of various natural compounds.

#### 3.1.6. Synergistic Effects and Combinations

Several studies have demonstrated enhanced efficacy when combining natural products. For example, an extract of *Taxus chinensis* var. *mairei* (AETC) potentiates the efficacy of osimertinib in overcoming resistance by targeting the ERK1/2/SREBP-2/HMGCR pathway and modulating cholesterol biosynthesis in cancer cells [28]. Furthermore, quercetin, when combined with simvastatin, exhibits a synergistic effect on HMGCR expression, suggesting enhanced therapeutic benefits [23]. These findings highlight the promising potential of combination therapies involving natural products.

**Table 1:** Natural products modulating HMGCR in hyperlipidemia/dyslipidemia.

Natural Product	Primary Bioactive Components	Dosage, Solvent for Extraction	Model System	HMGCR Effect	Proposed Mechanism	Reference
*Coreopsis tinctoria* Nutt. Extract	Luteolin, Marein, Naringenin, Chlorogenic Acid	Luteolin: 30 µM, marein: 10 µM, chlorogenic acid: 300 µM, naringenin: 200 µM	HepG2 cells (OA-induced)	Expression Downregulation	SREBP inhibition.	[18]
Cocoa Shell Ingredients (CSF/CSE)	Phenolic Compounds, Dietary Fiber	phenolic compounds (CSE: 271.4 mg 100 g^−1^, CSF: 43.8 mg 100 g^−1^), flour and aqueous extract	In vitro, hepG2 cells	Activity Inhibition	HMGCR inhibition may be linked to the phenolic compounds and dietary fiber. These compounds could interact with the HMGCR active site, reducing its activity.	[29]
Sanhua Jiangzhi Granules (SJG)	Complex Mixture of TCM Constituents	10 μg/mL, methanol and water in a 4:1 ratio	Rat model (HF Diet)	Expression Downregulation	PPAR signaling pathway activation.	[20]
Quercetin (from Buckwheat)	Quercetin	25, 50, 75, 100, 150, and 200 µg/mL, hexane, ethylacetate, and methanol	HepG2 cells	Expression Downregulation	Not fully elucidated; synergistic with simvastatin.	[23]
Forest Onion Extract (FOE)	Multiple—includes peptides	35, 70, 105, 140, and 175 µg/mL, 96% ethanol	3T3-L1 preadipocytes	Expression Downregulation	Inhibition of MAPK8, PPARG, HMGCR, CPT-1, and GLP-1 protein expressions.	[19]
*Lactiplantibacillus plantarum* SDJ09	Cell Extracts, Metabolites, Heat-Inactivated Cells	30 μg/mL, sterile water	HepG2 cells	Expression Downregulation	Reduction in lipid synthesis, upregulation of cholesterol excretion.	[30]
*Alpiniae oxyphyllae* Fructus (AOF)	Stigmasterol	0, 1, 2, 4, 8, 16 µM	Cell experiments	N/A (not directly investigated the effect of AOF or its components on HMGCR expression or activity levels)	Upregulated the expression levels of ESR1 and PPARG to exert an anti-HUA effect.	[31]
*Taxus chinensis* var. *mairei* (AETC)	Active Compound and Osimertinib combination	0.03 to 2 mg/mL, double-distilled water	Osimertinib-resistant cells and xenograft tumors in nude mice	Expression Downregulation	ERK/SREBP-2/HMGCR-mediated cholesterol biosynthesis and ROS levels.	[28]
Theabrownin from Qingzhuan tea (QTB)	Theabrownin extracted from Qingzhuan tea	180 or 360 mg/kg/d, absolute ethanol, then distilled water	HFD-induced mice	Expression Downregulation	Upregulate the expression of *ATGL*, *PPARα*, *FFAR2* and *FFAR3*, and inhibit the expression of *LXRα*, *SREBP-1c*, *FAS* and *HMGCR* genes.	[32]
Sinapic Acid	A Natural Source of Simple Phenolic Acids	0.03%	HFD-induced obesity hamster	Expression Downregulation	Egulationg the activities of PPAR-γ, CPT-1, and CYP7A1.	[24]
DI-HET, hydroethanolic extract from *Dillenia indica* leaf	Naringenin, Catechin, Epicatechin, Shikimic Acid, Syringic Acid, Vanillic Acid, and Kaempferol	5, 10, 20, 50, 100, 200, and 400 μg/mL, hydroethanolic extract	In vitro, HepG2 cells	Expression Downregulation	Activation of the SIRT-1/p-LKB-1/AMPK signaling pathway.	[33]
*Protium heptaphyllum* Gum Resin Extract (PHE)	Acidic Tetra- and Pentacyclic Triterpenoids	10–200 µg mL^−1^, hydroalcoholic extraction process	Hepatocytes	Expression Downregulation, Activity Inhibition	Reduce cholesterol production and regulate the expression of proteins involved in its metabolism.	[34]
Virgin Camellia Seed Oil	Active Compounds in Vegetable Oil	1.5 g/kg, squeezed using mall-pressed technologies	Sprague Dawley (SD) rats	Expression Downregulation	Modulating the AMPK-SREBP-signaling pathway.	[35]

Abbreviations: HepG2, hepatocellular carcinoma cell line; OA, oleic acid; SREBP, sterol regulatory element-binding protein; CSF/CSE, two cocoa shell ingredients, a flour (CSF) and an aqueous extract (CSE); PPAR, peroxisome proliferator-activated receptors; MAPK8, mitogen-activated protein kinase 8; CPT-1, carnitine palmitoyltransferase 1; GLP-1, glucagon-like peptide 1; ESR1, estrogen receptor 1; HUA, hyperuricemia; ERK, extracellular signal regulated kinase; ROS, reactive oxygen species; HFD, high-fat diet; ATGL, adipose triglyceride lipase; FFAR, free fatty acid receptors; *LXRα*, liver X receptor α; FAS, fatty acid synthase; CYP7A1, cholesterol 7α-hydroxylase; SIRT-1, Sirtuin 1; p-LKB-1, phospho-liver kinase B1; AMPK, AMP-activated protein kinase.

### 3.2. Cardiovascular Diseases (Also Summarized in Table 2)

#### 3.2.1. Targeting HMGCR in Atherosclerosis

Atherosclerosis, a major contributor to cardiovascular disease (CVD), is characterized by the accumulation of plaque within arterial walls. Dysregulation of lipid metabolism, resulting in elevated cholesterol levels, represents a critical step in the pathogenesis of atherosclerosis [36]. HMGCR, the rate-limiting enzyme in cholesterol biosynthesis, has long been recognized as a therapeutic target in this context. While statins are commonly employed to inhibit HMGCR, natural products are under investigation for their potential to modulate HMGCR activity, with the aim of achieving comparable therapeutic benefits while potentially mitigating adverse side effects.

#### 3.2.2. Modulation of HMGCR and Lipid Profiles by Natural Products

Several natural products have demonstrated promising effects on lipid profiles and HMGCR expression in models of cardiovascular disease (CVD). Tetrahydroxy stilbene glucoside (TSG) has shown potential in the treatment of atherosclerosis (AS). Specifically, TSG significantly restores the expression of fatty acid metabolism-related genes (*Srepb-1c*, *Fasn*, *Scd1*, *Gpat1*, *Dgat1*, *Pparα*, and *Cpt1α*) and regulates the expression levels of genes involved in cholesterol metabolism (*Srebp2*, *Hmgcr*, *Ldlr*, *Acat1*, *Acat2*, and *Cyp7a1*) that are dysregulated in association with lipid metabolism [36]. Furthermore, *Arctium lappa* leaves exhibit anti-atherosclerotic activity, although this particular study did not directly measure HMGCR levels [37]. In another animal study, flaxseed oil containing α-linolenic acid ester of plant sterol improved atherosclerosis in ApoE-deficient mice, which was associated with modulatory effects on the expression levels of genes involved in lipid metabolism, including *PPARα*, *HMGCR*, and *SREBPs* [38].

#### 3.2.3. Novel HMGCR Degraders for Enhanced Statin Therapy

A recent study has identified schipenindolene A (Spid A), a potent HMGCR degrader isolated from a fungal endophyte, as a promising agent for the treatment of cardiovascular disease (CVD). Unlike statins, which inhibit HMGCR activity, Spid A promotes the degradation of the HMGCR protein, reducing its levels to near-basal levels, even in the presence of statins [39]. This unique mechanism of action suggests that Spid A could be used in combination with statins to enhance their efficacy and potentially reduce side effects by counteracting the compensatory upregulation of HMGCR and lipogenesis enzymes that can occur in response to statin therapy.

#### 3.2.4. Black Elderberry Extract May Improve HDL Function

While not directly targeting HMGCR, the consumption of black elderberry extract has been shown to significantly lower serum chemokine (C-C motif) ligand 2 (CCL2) levels in comparison to control-fed mice. Importantly, significant reductions in the total cholesterol content of the aorta were observed in the black elderberry extract (BEE)-fed mice, indicating a decrease in atherosclerosis progression [40]. This study suggests that black elderberry may have the potential to modulate HDL dysfunction associated with chronic inflammation by influencing hepatic gene expression.

#### 3.2.5. Influence of the Intestinal Flora on Atherosclerosis

Several studies have demonstrated the beneficial effects of functional foods on improving cholesterol levels. A key factor contributing to these effects may be linked to the impact of environmental bacteria. Environmental bacteria can influence host hepatic inflammation and lipid distribution in the context of high-fat diets, with varying effects depending on the specific fat type consumed [41].

**Table 2:** Natural products targeting HMGCR in cardiovascular diseases.

Natural Product	Primary Bioactive Components	Dosage, Solvent for Extraction	Model System	HMGCR Effect	Proposed Mechanism	Reference
Tetrahydroxy stilbene glucoside (TSG)	N/A	100 mg/kg/day	ApoE^−/−^ mice	Expression Downregulation	Restores the expression of fatty acid metabolism-related genes.	[36]
Schipenindolene A (Spid A)	Indole diterpenoid	0.01–20 μM, trace fermented extract	In vitro cell culture	Protein Degradation	ERAD pathway activation.	[39]
*Arctium lappa* leaves	Various (unspecified)	12.5, 25, 50, 100, 200, and 400 μg/mL, 70% ethanol	Network pharmacology, in vitro and in vivo models	Did not directly measure HMGCR level	AMPK-mediated PPARG/*LXRα* pathway.	[37]
Flaxseed oil	A-linolenic acid ester of PS (ALA-PS)	flaxseed oil: 5% (*w*/*w*), ALA-PS: 3.3% (*w*/*w*)	ApoE-KO mice	Expression Downregulation	Modulatory effects on the expression levels of genes involved in lipid metabolism, including *PPARα*, *HMGCR*, and *SREBPs*.	[38]
Theabrownin from Qingzhuan tea (QTB)	Theabrownin extracted from Qingzhuan tea	180 or 360 mg/kg/d, absolute ethanol, then distilled water	HFD-induced mice	Expression Downregulation	Possible to act as a prebiotic to prevent MASLD. Reduces the expression of the HMGCR.	[32]

Abbreviations: ERAD, endoplasmic reticulum-associated degradation; AMPK, AMP-activated protein kinase; PPAR, peroxisome proliferator-activated receptors; *LXRα*, liver X receptor α; SREBP, sterol regulatory element-binding protein; HFD, high-fat diet; MASLD, metabolic dysfunction-associated steatosis liver disease.

### 3.3. Cancer (Also Summarized in Table 3)

#### 3.3.1. Targeting Cholesterol Synthesis in Cancer Therapy

The Role of HMGCR: Aberrant lipid metabolism, specifically the upregulation of cholesterol synthesis, has emerged as a characteristic feature of cancer cells, contributing to their growth, proliferation, and metastasis [42]. This metabolic rewiring makes enzymes involved in cholesterol synthesis, such as HMGCR, attractive therapeutic targets. Natural products represent a diverse source of compounds with the potential to modulate HMGCR activity, thereby disrupting cholesterol synthesis and impacting cancer cell viability. Recent studies have investigated the mechanisms through which natural products target HMGCR in various cancer types, providing insights into novel therapeutic strategies.

**Table 3:** Natural products targeting HMGCR in cancer.

Natural Product	Dosage, Solvent for Extraction	Cancer Type	HMGCR Effect	Proposed Mechanism	Reference
Cepharanthine (CE)	0.1–20 μM	Small Cell Lung Cancer (SCLC)	Expression Downregulation	Inhibition of cholesterol synthesis, direct binding to HMGCR and other enzymes	[43]
*Taxus chinensis* var. *mairei* (AETC)	0.03 to 2 mg/mL, double-distilled water	EGFR-mutant NSCLC	Expression Downregulation	Regulation of ERK1/2, inhibition of cholesterol biosynthesis	[28]
Gypenoside L	10 mg/kg or 20 mg/kg, 95% ethanol fraction	Hepatocellular Carcinoma (HCC)	Expression Downregulation	Targeting the SREBP2-HMGCS1 axis, regulating the mevalonate pathway	[44]
Carotenoids from *Spondias mombin*	100 mg/kg and 200 mg/kg, n-hexane/acetone 1:1 (*v*/*v*)	Breast Cancer	Expression Downregulation	Hydrophobic interactions with key residues within the catalytic domain of HMGCR	[45]
Chinese Red Yeast Rice (RYR)	0–300 μg/mL, methylene chloride	Prostate Cancer, Colon Cancer	No Significant Influence on Expression	Mechanisms beyond HMGCR are likely involved (related to pigments)	[46,47]

Abbreviations: EGFR, epidermal growth factor receptor; SREBP, sterol regulatory element-binding protein; ERK, extracellular signal regulated kinase; HMGCS1, 3-hydroxy-3-methylglutaryl-CoA synthase 1.

#### 3.3.2. Cepharanthine as a Small Cell Lung Cancer Inhibitor via HMGCR Modulation

A notable example is the identification of Cepharanthine (CE), an alkaloid isolated from plants of the genus *Stephania*, as a promising inhibitor of small cell lung cancer (SCLC). Integrated network pharmacology, RNA sequencing, and experimental validation studies have revealed that CE inhibits cholesterol synthesis in SCLC cells by downregulating key enzymes, including HMGCR, HMGCS1, IDI1, FDFT1, and SQLE [43]. Molecular docking studies further confirmed the binding of CE to these enzymes, suggesting a direct interaction. The cohesive docking energy results (kcal/mol) were as follows: HMGCS1: −7.8408, HMGCR: −7.9090, IDI1: −6.7217, FDFT1: −8.6832, and SQLE: −8.2930. Notably, high expression levels of *HMGCS1*, *HMGCR*, and *IDI1* in SCLC cells correlated with poor prognosis, and silencing these genes significantly suppressed SCLC cell proliferation. These findings highlight the potential of CE as a therapeutic agent for SCLC, acting through the suppression of cholesterol synthesis.

#### 3.3.3. *Taxus chinensis* var. *mairei* Extract (AETC) to Overcome Osimertinib Resistance in Non-Small Cell Lung Cancer (NSCLC)

Resistance to osimertinib has been linked to alterations in cholesterol biosynthesis. AETC can enhance the sensitivity of NSCLC cells to osimertinib by targeting the ERK1/2/SREBP-2/HMGCR pathway and modulating cholesterol biosynthesis [28]. The study demonstrates that AETC enhances the efficacy of osimertinib in overcoming resistance through this mechanism.

#### 3.3.4. Gypenoside L as a Hepatocellular Carcinoma Inhibitor via HMGCR Regulation

Gypenoside L (Gyp L), a compound derived from *Gynostemma pentaphyllum*, has demonstrated inhibitory effects on hepatocellular carcinoma (HCC) cells by reducing cholesterol and triglyceride levels and targeting the mevalonate (MVA) pathway, a critical route for cholesterol biosynthesis [44]. Gyp L treatment significantly decreased the expression of HMGCS1 and HMGCR in HCC cells, leading to significant alterations in the cholesterol metabolism pathway within HCC cells and enhancement of anticancer immune responses.

#### 3.3.5. Carotenoids from *Spondias mombin* Demonstrate HMGCR Inhibition

This study provided in silico and in vivo evidence demonstrating that carotenoids from *Spondias mombin* inhibit HMGCR and exert anti-tumorigenic effects. The in silico docking experiments predicted that three carotenoids from *S. mombin* (beta-carotene-15,15′-epoxide, astaxanthin, and 7,7′,8,8′-tetrahydro-β-β-carotene) could directly interact with HMGCR. The docking scores (kcal/mol) were −6.3, −5.9, and −6.1, respectively. Furthermore, there were 4 hydrophobic interactions (met-557, asn-658, val-593, ala-682) for 7,7′,8,8′-tetrahydro-β-β-carotene, 5 hydrophobic interactions (met-658, asn-658, lys-691, ala-682, leu-681) for astaxanthin, and 4 hydrophobic interactions (met-655, asn-658, lys-691, val-683) for beta-carotene-15,15′-epoxide within the catalytic portion [45].

#### 3.3.6. Chinese Red Yeast Rice (RYR)

This research compared the effects of Chinese red yeast rice and lovastatin (LV) on prostate cancer cells. While LV upregulates *HMGCR* gene expression, RYR does not. This suggests that the mechanism by which RYR affects cholesterol synthesis may involve pathways other than direct HMGCR modulation [46].

These studies underscore the potential of natural products in targeting HMGCR for cancer therapy. However, they also emphasize the complexity of their mechanisms of action, which often involve multiple targets and signaling pathways.

## 4. Mechanisms of HMGCR Modulation by Natural Products

### 4.1. HMGCR as a Target

Network pharmacology has become a valuable tool in drug discovery, frequently identifying HMGCR as a potential therapeutic target across a broad range of diseases. For example, in the context of hyperlipidemia, both Sanhua Jiangzhi granules and a formula containing *Curcuma xanthorrhiza* have been found to target HMGCR, suggesting a mechanism for regulating lipid metabolism [20,48]. Studies investigating herbal medicines for alcoholic liver disease and cholesterol gallstones have also implicated HMGCR, with molecular docking studies revealing strong binding affinities between compounds such as hydroxysafflor yellow A and naringenin with HMGCR, indicating potential therapeutic avenues [49,50]. This approach extends to other diseases, as seen in studies on nephrotic syndrome using Zhuling Decoction and small cell lung cancer using Cepharanthine, where HMGCR was identified as a hub target involved in the PI3K-Akt, Ras, MAPK, and cholesterol metabolism pathways, respectively [43,51]. Even in the context of hyperuricemia, *Alpiniae oxyphyllae* Fructus was predicted to have therapeutic effects by binding to HMGCR, suggesting its broad relevance in various metabolic and disease contexts [31]. These studies emphasize the value of network pharmacology in elucidating potential therapeutic targets and mechanisms of action for diverse compounds and diseases.

**Figure 4:**
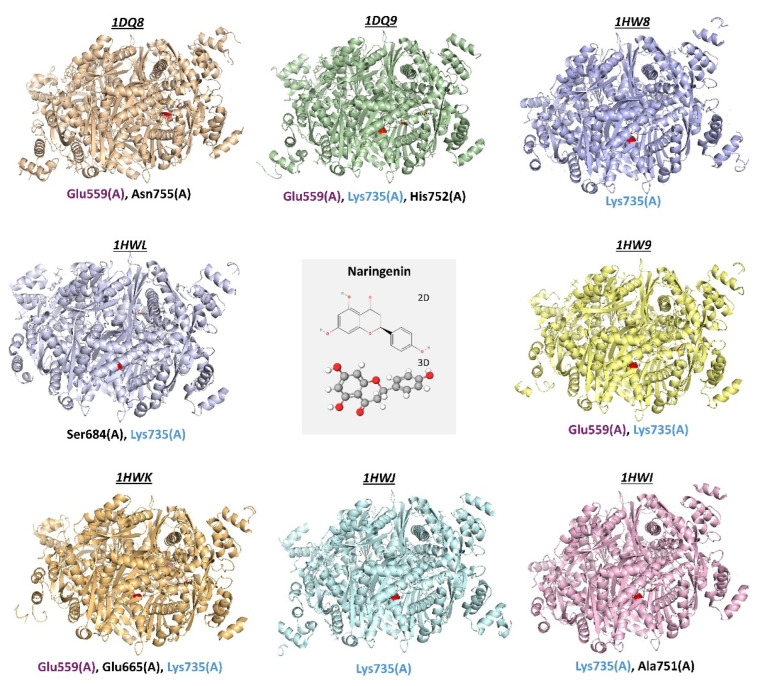
Molecular docking interactions between naringenin and HMGCR [50,53,54]. The Protein Data Bank (PDB) ID for each HMGCR structure is indicated above the corresponding protein depiction. Crucial amino acid residues involved in the interaction are highlighted in red below each protein structure. Glu559 and Lys735 represent common amino acids involved in the interactions with naringenin across the different PDB structures. The 2D and 3D structures of naringenin were retrieved from the PubChem database.

Molecular docking simulations are increasingly utilized in conjunction with network pharmacology to investigate potential interactions between bioactive compounds and target proteins, offering insights into possible mechanisms of action. For example, in the study of *Alpiniae oxyphyllae* Fructus against hyperuricemia, molecular docking suggested that active ingredients could bind to targets such as PPARG, ESR1, PTGS2, and HMGCR, potentially mediating therapeutic effects on immune and inflammatory responses. The average binding energy (kcal/mol) of stigmasterol, sitosterol, daucosterol, and sitosteryl palmitate for HMGCR was −7.49, −5.01, −2.52, and −0.89, respectively [31]. Similarly, hydroxysafflor yellow A (HSYA) in the context of alcoholic liver disease, and apigenin triacetate for hypercholesterolemia-associated neurodegeneration, exhibited strong binding affinities toward HMGCR, PPARA, and PPARG through molecular docking, hinting at their roles in mitigating lipid accumulation and inflammation [49,52]. Moreover, in the treatment of cholesterol gallstones, molecular docking results confirmed the interaction between genes and naringenin, an active ingredient in Shugan Lidan Xiaoshi Granules (SLXGs). The binding energies of molecular docking between naringenin and UGT1A1, HMGCR, and SOAT2 were all below −7 [50]. Furthermore, studies on *Gynostemma pentaphyllum* and a formula containing *Curcuma xanthorrhiza* also employed molecular docking to identify potential interactions between their active components and targets such as HMGCR and PPARs, suggesting mechanisms for their anti-obesity and antihyperlipidemic effects [26,48]. The vina software docking scores of compounds in the *Curcuma xanthorrhiza* on HMGCR were as follows: β-sitosterol (−5.9), Bisdesmethoxycurcumin (−6.5), Cucurbitacin D (−7.4), Cucurbitacin E (−7.3), Myricetin (−8.0), Phloretin (−7.1), Quercitrin (−8.4), and Rutin (−8.9) [48]. Even in the case of Zhuling Decoction against nephrotic syndrome, molecular docking indicated high binding activities between its compounds and HMGCR, HSD11B1, and NOS2 [51]. These findings collectively highlight the utility of molecular docking simulations in exploring potential interactions between bioactive compounds and target proteins, contributing to a deeper understanding of their pharmacological mechanisms. Figure 4 summarizes the molecular interactive results between naringenin and HMGCR from several studies employing molecular docking as an investigative tool.

### 4.2. HMGCR Activity Inhibition

Direct inhibition of HMGCR activity by specific natural compounds is a well-established mechanism for cholesterol reduction, often mimicking the mechanism of action of statin drugs. Studies have demonstrated that various extracts and isolated compounds can directly target HMGCR, reducing its activity and subsequently lowering cholesterol levels. For instance, an in vitro study revealed that extracts from Citrus Tacle^®^, rich in polyphenols like naringenin and hesperetin, exhibited HMGCR inhibitory activity, with molecular docking suggesting that these compounds can bind to the enzyme with affinities comparable to those of statins [53]. Similarly, *Mikania micrantha* extract demonstrated the ability to inhibit HMGCR activity in vivo, leading to a reduction in cholesterol levels in high cholesterol-fed rats [55]. A study on tomato juice found that its consumption led to a significant reduction in HMGCR activity in rats, with molecular modeling suggesting that compounds such as lycopene can bind to the active site of the enzyme [54]. Furthermore, cocoa shell ingredients, following simulated digestion, showed enhanced HMGCR inhibitory activity in vitro, contributing to their observed lipid-lowering effects in HepG2 cells [29]. These studies provide compelling evidence for the direct inhibition of HMGCR activity by specific natural compounds, offering a potential avenue for developing natural alternatives for managing hypercholesterolemia.

### 4.3. HMGCR Expression Regulation (Also Summarized in Table 4)

HMGCR expression, at both the mRNA and protein levels, represents a key regulatory point in cholesterol homeostasis, and various natural products have been shown to influence it. These effects often contribute to the overall lipid-lowering properties of these compounds. This regulation can occur through multiple mechanisms, frequently involving transcription factors and signaling pathways.

#### 4.3.1. Downregulation of HMGCR Expression

Numerous studies have demonstrated the ability of natural compounds to reduce HMGCR expression. For instance, *Coreopsis tinctoria* Nutt. extracts (luteolin, marein, NGN, and CQA) downregulated *HMGCR* mRNA expression in oleic acid (OA)-treated HepG2 cells [18]. Similarly, theabrownin from Qingzhuan tea inhibited *HMGCR* gene expression by regulating the AMPK-PPAR pathway [32], and fermented *Rhus verniciflua* Stokes extract decreased both mRNA and protein levels of HMGCR in oleic acid-induced HepG2 cells Via AMPK upregulation [27]. Zexie Tang, targeting the FKBP38/mTOR/SREBPs pathway, also reduced HMGCR expression [56].

#### 4.3.2. SREBP-2 Modulation

Sterol regulatory element-binding protein-2 (SREBP-2) is a crucial transcription factor that regulates HMGCR. Natural products often exert their effects by modulating this pathway. Flaxseed oil (FO), a dietary oil rich in α-linolenic acid, decreased the protein expression of SREBP2, HMGCR, and LDLR, while increasing the expression of CYP7A1 [57]. Unripe *Rubus coreanus* extract and ellagic acid suppressed the nuclear translocation and activation of SREBP-2 [58]. In addition, extracts from *Taxus chinensis* var. *Mairei* downregulate key regulators of cholesterol biosynthesis by regulating ERK1/2, inhibiting the endogenous synthesis rate of cholesterol Via ERK/SREBP-2/HMGCR-mediated cholesterol biosynthesis [28].

#### 4.3.3. AMPK Activation

Certain natural compounds influence HMGCR expression through the activation of AMP-activated protein kinase (AMPK). GINST, a hydrolyzed ginseng extract, inhibits cholesterol synthesis in HepG2 cells by decreasing HMGCR expression via AMPKα activation [59]. Furthermore, the inhibitory effect of GINST on HMGCR expression was reversed when cells were treated with dorsomorphin, an AMPK inhibitor. This suggests that GINST decreases HMGCR expression via AMPK activation. Additionally, a flavonoid-rich extract from *Paulownia fortunei* flowers attenuated diet-induced hyperlipidemia by activating the AMPK pathway [60].

#### 4.3.4. Other Mechanisms

Alternative mechanisms include the direct degradation of HMGCR protein, as observed with schipenindolene A (Spid A), which activated the endoplasmic reticulum-associated degradation (ERAD) pathway to enhance HMGCR degradation [39]. Spid A showed a synergistic effect with statins in lowering cholesterol levels, potentially reducing statin-induced side effects by counteracting the compensatory upregulation of HMGCR and lipogenesis enzymes caused by statin therapy. Conversely, certain natural products upregulate HMGCR expression, such as *Celastrus orbiculatus* Thunb. (upregulated mRNA abundance) [61], or black raspberry extract (tended to enhance CYP7A1 and ABCG5 expression) [62].

Understanding the mechanisms by which natural products regulate HMGCR expression is essential for developing effective strategies to manage cholesterol levels and prevent related diseases. The modulation of these pathways provides a multifaceted approach to addressing lipid metabolism, potentially offering benefits beyond those achieved through direct HMGCR inhibition alone.

**Table 4:** Table summarizing HMGCR expression regulation by natural products.

Natural Product/Extract	Effect on HMGCR Expression	Mechanism	Reference
*Coreopsis tinctoria* Nutt. extracts (luteolin, marein, NGN, CQA)	Downregulation	Reduce OA-induced oxidative stress and lipid accumulation	[18]
Aqueous extract of *Taxus chinensis* var. *Mairei*	Downregulation	ERK/SREBP-2/HMGCR-mediated cholesterol biosynthesis	[28]
Flavonoid-rich extract of *Paulownia fortunei* Flowers	Decrease	AMPK pathway	[60]
GINST (hydrolyzed ginseng extract)	Decrease	AMPKα activation	[59]
*Sargassum fusiforme* polysaccharide (SFP) co-administered with low-dose acarbose	Decrease in the expression of the *HMGCR* gene	Affecting the expression of *HMGCR* and *SREBP-1c* genes, restraining liver fat accumulation	[63]
Flaxseed oil (FO), a dietary oil rich in α-linolenic acid	Decreased protein expression	Decreased the protein expression of SREBP2, HMGCR, and LDLR while increasing the expression of CYP7A1	[57]
Annatto-derived tocotrienol	Downregulated *HMGCR* gene expression	Downregulating *HMGCR* gene expression and inhibiting RhoA activation, leading to increased BMP-2 protein	[64]
*Celastrus orbiculatus* Thunb.	Upregulated *HMGCR* mRNA	LDL-R, SR-B1, CYP7A1	[61]
Black raspberry extract	Downregulated *Srebf2* and *Hmgcr* expressions	Upregulate and enhance *Cyp7a1* and *Abcg5* expressions	[62]

Abbreviations: NGN, naringenin; CQA, chlorogenic acid; OA, oleic acid; ERK, extracellular signal regulated kinase; SREBP, sterol regulatory element-binding protein; AMPK, AMP-activated protein kinase; LDLR, LDL receptor; CYP7A1, cholesterol 7α-hydroxylase; BMP-2, bone morphogenetic protein-2; SR-B1, scavenger receptor class B type 1; Abcg5, ATP-binding cassette transporter G5.

### 4.4. Signaling Pathways Involvement (Also Summarized in Table 5)

Natural products frequently regulate HMGCR through diverse signaling pathways, influencing cholesterol synthesis and lipid metabolism. Several studies have highlighted the involvement of SREBP, PPAR, AMPK, and ERK1/2 pathways in this regulatory process, with the PI3K-Akt and mTOR signaling pathways also playing significant roles.

#### 4.4.1. SREBP Pathway

Certain natural products influence cholesterol synthesis by modulating the sterol regulatory element-binding protein (SREBP) pathway, a key regulator of lipid metabolism. For example, the aqueous extract of *Taxus chinensis* var. *Mairei* was found to downregulate key regulators of cholesterol biosynthesis by regulating ERK1/2, inhibiting the endogenous synthesis rate of cholesterol via ERK/SREBP-2/HMGCR-mediated cholesterol biosynthesis [28]. Similarly, unripe *Rubus coreanus* extract and ellagic acid suppressed the nuclear translocation and activation of SREBP-2, a key transcription factor in cholesterol biosynthesis [58]. In addition, they also activated AMPK, which in turn inhibited HMGCR activity via inhibitory phosphorylation. These provided dual mechanisms by which unripe *Rubus coreanus* extract and ellagic acid might lower cholesterol levels.

#### 4.4.2. PPAR Pathway

The peroxisome proliferator-activated receptor (PPAR) pathway is another critical target for natural products that influence lipid metabolism. Sanhua Jiangzhi Granules activated the PPAR signaling pathway, leading to decreased body weight, lowered blood lipid levels, reduced hepatic total cholesterol (TC) and triglycerides (TGs), and improved liver pathology [20]. Additionally, red raspberry extract was shown to ameliorate hyperlipidemia in high-fat-diet-induced mice through the PPAR signaling pathway [65].

#### 4.4.3. AMPK Pathway

Numerous natural products activate AMP-activated protein kinase (AMPK), a central regulator of energy homeostasis, leading to the inhibition of HMGCR. Virgin camellia seed oil improved glycolipid metabolism by modulating the AMPK-SREBP signaling pathway [35]. A flavonoid-rich extract from *Paulownia fortunei* flowers (EPFs) also attenuated diet-induced hyperlipidemia through the AMPK pathway [60]. EPF increased AMPK phosphorylation, and this effect was blocked by compound C, an AMPK inhibitor. This suggests that AMPK activation is crucial to EPF’s effects on HMGCR and overall lipid metabolism. Furthermore, strawberry extract decreased cholesterol, LDL, and triglycerides by stimulating AMPK expression, which led to the inhibition of HMGCR [66]. The observed reduction in HMGCR activity was due to the activation of AMPK by the strawberry extract. AMPK activation, in turn, led to the phosphorylation and inactivation of ACC (acetyl-CoA carboxylase), another key regulator of fatty acid synthesis. Treatment with compound C, an AMPK inhibitor, reversed the inhibitory effect of the strawberry extract on HMGCR, confirming the central role of AMPK in this process.

#### 4.4.4. ERK1/2 Pathway

An aqueous extract of *Taxus chinensis* var. *Mairei* overcomes resistance to osimertinib in EGFR-mutant non-small-cell lung cancer by suppressing ERK1/2-related cholesterol biosynthesis [28]. A strong positive correlation was observed between ERK1/2 expression and the expression levels of SREBP-2 and HMGCR. Knockdown of ERK1/2 decreased SREBP-2 and HMGCR expression, while overexpression increased them. This further supports the link between ERK1/2 activation and increased cholesterol biosynthesis in osimertinib resistance.

#### 4.4.5. PI3K-Akt Pathway

Baicalin and wogonoside, components of the Gandi capsule, can ameliorate high glucose (HG)-induced podocyte damage by influencing the AMPK and PI3K-AKT signaling pathways [67]. These findings suggest that baicalin and wogonoside influence the PI3K-AKT and AMPK signaling pathways by binding to HNF4A, HMGCR, JAK3, and SIRT1.

#### 4.4.6. mTOR Pathway

Zexie Tang, by targeting the FKBP38/mTOR/SREBPs pathway, improves hyperlipidemia, suggesting a potential regulatory mechanism involving its active compounds [56]. Zexie Tang inhibited SREBP expression and activity, and consequently reduced the expression of SREBP target genes, including HMGCR, leading to decreased cholesterol synthesis.

These examples illustrate the complex interplay between natural products and cellular signaling pathways in the regulation of HMGCR and cholesterol metabolism, underscoring the potential of these compounds for the development of novel therapeutic strategies for hyperlipidemia and related disorders.

**Table 5:** Table summarizing the involvement of signaling pathways.

Natural Product or Extract	Signaling Pathway(s) Involved	Effect on HMGCR	Reference
Sanhua Jiangzhi Granules	PPAR	Downregulation	[20]
*Taxus chinensis* var. *Mairei* Extract	ERK/SREBP-2	Downregulation	[28]
Virgin Camellia Seed Oil	AMPK-SREBP	Downregulation	[35]
Unripe *Rubus coreanus* Extract and Ellagic acid	AMPK, SREBP-2	Reduce HMGCR Activity	[58]
Red Raspberry Extract	PPAR	Downregulation	[65]
Flavonoid-rich Extract of *Paulownia fortunei* Flowers	AMPK	Decrease	[60]
Strawberry Methanolic Extract	AMPK	Inhibition	[66]
Gandi capsule	AMPK, PI3K-Akt	HMGCR identified as a potential target	[67]
Zexie Tang	FKBP38/mTOR/SREBPs	Downregulation	[56]

Abbreviations: PPAR, peroxisome proliferator-activated receptors; ERK, extracellular signal regulated kinase; SREBP, sterol regulatory element-binding protein; AMPK, AMP-activated protein kinase; PI3K-Akt, PI3K (phosphatidylinositol 3-kinase) and Akt (protein kinase B); FKBP38, FK506-binding protein 8; mTOR, mammalian target of rapamycin.

### 4.5. Comparison or Combination with Statins or Other Therapies (Also Summarized in Table 6)

A comparison of the potency and efficacy of natural inhibitors to those of statins presents a complex landscape. While statins generally exhibit higher potency in directly inhibiting HMGCR, certain natural compounds and extracts demonstrate comparable or even synergistic effects through alternative mechanisms. Moreover, natural products often provide additional benefits beyond cholesterol reduction, potentially mitigating some of the adverse effects associated with statin use.

One area where natural products demonstrate particular promise is in synergistic combinations with statins. For instance, in vitro experiments on HepG2 cells treated with quercetin (derived from buckwheat) revealed a synergistic action in modulating cholesterol levels when combined with simvastatin [23]. This combination led to a further marked increase in *CYP7A1* gene expression and a more significant reduction in *HMGCR* gene expression. This suggests that lower doses of statins, in combination with natural compounds, could achieve comparable therapeutic effects while minimizing adverse reactions. Evaluating synergy between natural products or between natural products and conventional drugs requires rigorous methodologies to distinguish true synergistic effects from additive or antagonistic interactions. Several approaches are commonly employed, including isobolographic analysis, which assesses whether the combination of two agents produces a greater-than-additive effect based on their individual dose–response curves [68]. The combination index (CI) method, often used in drug discovery, quantifies the interaction between two agents, with CI values less than 1 indicating synergy, CI equal to 1 indicating additivity, and CI greater than 1 indicating antagonism [69]. Furthermore, response surface methodology can be used to model the combined effect of multiple agents across a range of concentrations, providing a comprehensive assessment of their interaction [70]. These methods provide a framework for characterizing and quantifying synergistic interactions, facilitating the rational design of combination therapies. Another example is the co-administration of *Sargassum fusiforme* polysaccharide (SFP) with a low dose of acarbose in type 2 diabetic rats, which mitigated diabetic symptoms and improved serum profiles, exhibiting superior anti-diabetic effects compared to acarbose treatment alone. This combination also improved insulin resistance, reduced kidney injuries, and restrained liver fat accumulation, affecting the expression of *HMGCR* and *SREBP-1c* genes [63].

However, direct comparisons of potency often favor statins. For example, an in vitro study using HepG2 cells found that the IC_50_ values for inhibiting HMGCR were 59.2 mg/L for a chlorogenic acid-enriched extract from *Eucommia ulmoides* leaves, 335.9 μmol/L for chlorogenic acid, and 10.5 μmol/L for simvastatin, highlighting the greater potency of the statin [71].

Despite the lower potency of individual natural compounds, certain extracts can exert significant cholesterol-lowering effects in vivo, sometimes approaching the efficacy of statins. For example, in hyperlipidemic hamsters, *Curcuma* oil at a dosage of 300 mg/kg exhibited an anti-hyperlipidemic effect comparable to that of ezetimibe, a standard cholesterol-lowering drug, by modulating *PPARα*, *LXRα*, and associated genes involved in lipid metabolism and transport [72]. Additionally, annatto-derived tocotrienol (AnTT) can suppress the mevalonate pathway by downregulating *HMGCR* gene expression in MC3T3-E1 cells [64].

It is crucial to consider the pleiotropic effects of natural products. While statins primarily target HMGCR, natural compounds may act on multiple pathways concurrently. Forest onion extract (FOE) inhibits the targeted protein expression of MAPK8, PPARG, HMGCR, CPT-1, and GLP-1 in vitro in 3T3-L1 mouse cells in a dose-dependent manner [19]. Red yeast rice (RYR), which contains monacolin K (identical to lovastatin), exhibits anticancer effects on colon cancer cells, and these effects were not reversed. This suggests that other components in RYR may affect intracellular signaling pathways differently from purified, crystallized lovastatin [47].

In conclusion, while natural inhibitors of HMGCR may not always match the direct potency of statins, they offer unique advantages, including synergistic potential, multi-targeting mechanisms, and potentially fewer adverse effects. These characteristics make them valuable options for the management of hyperlipidemia, particularly in combination therapies or for individuals seeking gentler, more holistic approaches to cholesterol control.

**Table 6:** Table comparing natural inhibitors and statins.

Natural Inhibitor/Extract	Potency Compared to Statins	Key Mechanisms	Reference
Quercetin	Synergistic with Simvastatin	Reduces HMGCR expression, increases CYP7A1 expression.	[23]
Chlorogenic Acid-Enriched Extract from *Eucommia ulmoides* leaves	Lower IC_50_ than Simvastatin	Increases *ABCA1*, *CYP7A1*, and *AMPKα2* mRNA expression, decreases *SREBP2.*	[71]
*Curcuma* Oil	Comparable to Ezetimibe (at high dose)	Modulates *PPARα*, *LXRα*, and associated genes in lipid metabolism and transport.	[72]
Forest Onion Extract	N/A	inhibits targeted protein expressions of MAPK8, PPARG, HMGCR, CPT-1, and GLP-1 in vitro in 3T3-L1 mouse cells.	[19]
Red Yeast Rice	N/A (In contrast to LV, neither RYR nor PF-RYR significantly altered the expression of HMGCR or SREBP-2)	Exhibit anticancer effects on colon cancer cells. May affect intracellular signaling pathways differently from purified crystallized LV.	[47]
*Sargassum fusiforme* polysaccharide (SFP)	N/A	Restores beneficial gut flora and activates IRS/PI3K/AKT signaling pathway (in combination with low-dose acarbose). Restrained liver fat accumulation via affecting the expression of *HMGCR* and *SREBP-1c* genes.	[63]

Abbreviations: CYP7A1, cholesterol 7α-hydroxylase; ABCA1, ATP-binding cassette transporter A1; AMPK, AMP-activated protein kinase; SREBP, sterol regulatory element-binding protein; PPAR, peroxisome proliferator-activated receptors; *LXRα*, liver X receptor α; MAPK8, mitogen-activated protein kinase 8; CPT-1, carnitine palmitoyltransferase 1; GLP-1, glucagon-like peptide 1; LV, lovastatin; PF-RYR, pigment-rich fraction of Chinese red yeast rice; IRS, insulin receptor substrate; PI3K/AKT, PI3K (phosphatidylinositol 3-kinase) and Akt (protein kinase B).

## 5. Challenges and Future Directions

### 5.1. Research Gaps in Natural HMGCR Modulators

The therapeutic potential of natural HMGCR modulators remains largely unrealized due to critical gaps in research. A scarcity of robust in vivo studies limits our understanding of their efficacy and safety in complex biological systems. A significant portion of research relies on in vitro data, neglecting key pharmacokinetic and pharmacodynamic considerations. Rigorous clinical trials are urgently needed to validate their clinical applicability and assess lipid-lowering effects, long-term safety profiles, and potential drug interactions. Further research is also required to optimize dosage regimens, formulations, and combination therapies. Addressing these knowledge gaps is crucial for successfully integrating natural HMGCR modulators into clinical practice for the management of dyslipidemia and cardiovascular disease.

### 5.2. Challenges in Translating In Vitro Data to Clinical Application

Translating in vitro HMGCR modulation data to clinical applications presents significant challenges. The discrepancy between controlled laboratory settings and the complex in vivo environment, encompassing metabolism, absorption, and bioavailability, significantly impacts a compound’s effectiveness [73,74]. In vitro studies often utilize isolated systems, inadequately reflecting whole-body physiology and inter-system interactions [75,76]. Moreover, the potential for synergistic or antagonistic effects when combining natural products with other medications is frequently overlooked, potentially leading to adverse interactions or reduced efficacy in humans [77,78]. While in vitro studies provide valuable preliminary information, rigorous preclinical and clinical validation is essential for safe and effective therapeutic translation. Translating promising preclinical findings with natural products into successful clinical applications presents numerous hurdles. Regulatory complexities, stemming from the poorly defined nature of many plant extracts and the lack of standardized quality control, pose a significant challenge [79]. The inherent variability in the composition of plant extracts due to factors such as geographical origin, growing conditions, and extraction methods introduces inconsistencies that complicate clinical trial design and data interpretation [80]. Furthermore, the use of combination therapies involving multiple natural products or natural products with conventional drugs adds another layer of complexity, requiring careful evaluation of potential synergistic or antagonistic effects and drug interactions [81]. Overcoming these challenges demands rigorous standardization of natural product preparations, comprehensive pharmacokinetic and pharmacodynamic studies, and innovative clinical trial designs to assess efficacy and safety in human populations.

### 5.3. Clinical Trial Suggestion

#### 5.3.1. Hyperlipidemia

For evaluating natural products in the treatment of hyperlipidemia, randomized controlled trials (RCTs) are essential. These trials should compare the natural product to a placebo or, ideally, to a standard-of-care statin therapy to assess relative efficacy [82]. Combination therapy RCTs, where the natural product is used in conjunction with a statin, can also be valuable to determine if there is an additive or synergistic effect on lipid lowering. Dose-ranging studies are necessary to establish the optimal dosage of the natural product. The primary endpoint in these trials should be the percentage reduction in LDL-C (low-density lipoprotein cholesterol) from baseline to a defined time point (e.g., 12 weeks) [83]. Secondary endpoints can include changes in other lipid parameters (HDL-C, triglycerides), safety assessments, and markers of liver function.

#### 5.3.2. Cardiovascular Disease

Clinical trials evaluating natural products for cardiovascular disease prevention or treatment should prioritize cardiovascular event reduction as the primary endpoint. This could include composite endpoints such as myocardial infarction, stroke, hospitalization for heart failure, or cardiovascular death [84]. RCTs comparing the natural product to placebo or standard therapies (e.g., antiplatelet agents, ACE inhibitors) are necessary. Given the multifactorial nature of cardiovascular disease, combination therapy trials may also be warranted. Furthermore, studies should include relevant biomarkers such as blood pressure, inflammatory markers (e.g., CRP, C-Reactive Protein), and measures of endothelial function as secondary endpoints. The trial duration should be sufficiently long to capture a meaningful number of cardiovascular events.

#### 5.3.3. Cancer

Clinical trials investigating natural products in cancer treatment should be designed to assess their impact on cancer progression and overall survival. RCTs are essential, comparing the natural product to placebo, standard chemotherapy, or as an adjunct to standard therapy (e.g., assessing whether a natural product can improve response rates or reduce side effects of chemotherapy) [85]. Dose-ranging studies are crucial to identify optimal dosages and minimize toxicity. The primary endpoint could be progression-free survival (PFS), defined as the time from randomization to disease progression or death, or overall survival (OS), defined as the time from randomization to death from any cause. Secondary endpoints can include objective response rate (ORR), quality of life measures, and changes in relevant biomarkers (e.g., tumor markers, immune cell populations).

### 5.4. Need for Mechanistic Elucidation

Comprehensive mechanistic elucidation of natural compounds targeting HMGCR is crucial given HMGCR’s central role in cholesterol biosynthesis and lipid management. While preliminary studies suggest effects on HMGCR activity, the underlying pathways and molecular interactions remain largely undefined [29,53,86]. A thorough understanding of these mechanisms is essential for establishing efficacy and safety. This includes elucidating how specific phytochemicals modulate HMGCR, impacting cholesterol metabolism, gene expression, and interactions with other metabolic pathways. Furthermore, pharmacokinetic characterization, determination of optimal dosing regimens, and assessment of side effect profiles are critical for developing these compounds as viable alternatives or adjuncts to statin therapy. Future research should integrate in vitro, in vivo, and translational approaches to achieve a holistic understanding that can inform therapeutic applications and ultimately improve patient outcomes.

### 5.5. Limitations in Natural Product Development

Natural products hold immense promise in drug discovery, yet their development faces significant limitations. These include low yields, structural complexity hindering synthesis, and challenges in identifying the active compounds responsible for observed biological effects. Furthermore, bioavailability issues and a lack of comprehensive pharmacological data often impede clinical translation. To address these limitations, strategies such as employing advanced extraction and purification techniques, utilizing semi-synthetic approaches to simplify structures, and leveraging metabolomics and bioactivity-guided fractionation to pinpoint active constituents are crucial. Additionally, improving bioavailability through formulation strategies and conducting thorough preclinical and clinical studies ais necessary to fully realize the therapeutic potential of natural products [87,88,89].

### 5.6. Bioavailability, Metabolism, and Safety Considerations

In addition, the complicated issues of bioavailability, metabolism, toxicity, and safety of natural products also need to be considered in future research and applications. For instance, naringenin, a flavanone found in citrus fruits, exhibits moderate bioavailability due to its limited water solubility and extensive first-pass metabolism. Following absorption, naringenin undergoes rapid glucuronidation and sulfation, primarily in the liver and intestine, leading to the formation of various metabolites with potentially altered bioactivity. Regarding safety, naringenin generally demonstrates low toxicity in preclinical studies, with reported adverse effects primarily at high doses. However, it can interact with certain drug-metabolizing enzymes, such as CYP3A4, potentially affecting the pharmacokinetics of co-administered medications. Therefore, while naringenin holds promise for various health benefits, further research is needed to fully elucidate its metabolic pathways, potential drug interactions, and long-term safety profile in humans [90].

### 5.7. Potential Interactions with Conventional Medications

The potential for interactions between natural products and conventional medications, such as statins, warrants careful consideration due to the risk of altered drug efficacy and adverse effects. Several natural products can modulate the activity of cytochrome P450 (CYP450) enzymes, a family of enzymes crucial for drug metabolism. For example, St. John’s Wort is a well-known inducer of CYP3A4, an enzyme responsible for metabolizing many statins, potentially leading to reduced statin plasma concentrations and diminished therapeutic effect [91]. Conversely, grapefruit juice inhibits CYP3A4, which can increase statin levels and elevate the risk of myopathy and rhabdomyolysis [92]. These examples highlight the clinical relevance of understanding potential herb–drug interactions and the importance of healthcare professionals being aware of patients’ use of natural products to avoid adverse outcomes [93].

### 5.8. Batch-to-Batch Variability Challenges

Batch-to-batch variability in the composition of natural products presents a critical challenge for clinical translation and the reproducibility of research findings [79]. Unlike synthetic pharmaceuticals with precisely defined chemical structures, natural products, particularly plant extracts, can exhibit significant variations in the concentration of their active components due to factors such as genetic variations, environmental conditions, harvesting practices, and extraction methods [80]. This variability complicates the establishment of consistent dosing regimens and can lead to inconsistent clinical outcomes. Therefore, standardization of natural product preparations based on the identification and quantification of key bioactive compounds is essential to ensure reproducibility and facilitate reliable clinical evaluation [94]. Such standardization efforts are crucial for bridging the gap between promising preclinical results and successful clinical applications.

### 5.9. Potential Risks of Natural Products

While many natural products are perceived as safe, it is crucial to recognize that they are not without potential risks, particularly with high-dose or long-term use. For instance, berberine, a compound found in several plants, has demonstrated promising effects on glucose and lipid metabolism; however, it can cause gastrointestinal side effects such as nausea, diarrhea, and abdominal discomfort in some individuals [95]. Red yeast rice, another popular natural product for cholesterol management, contains monacolin K, which is chemically identical to the statin drug lovastatin. Consequently, red yeast rice can produce similar side effects to statins, including myopathy, liver toxicity, and rhabdomyolysis, particularly in susceptible individuals or when taken in combination with other cholesterol-lowering medications [96]. These examples underscore the importance of careful risk-benefit assessments, appropriate dosage monitoring, and awareness of potential adverse effects and drug interactions when considering the use of natural products for therapeutic purposes.

### 5.10. Priorities for Future Research

Future research on natural HMGCR modulators should prioritize the investigation of novel compounds derived from diverse botanical sources, many of which remain uncharacterized with respect to lipid modulation. Comprehensive mechanistic studies are necessary to clarify the interactions of these compounds with HMGCR and other metabolic pathways, thereby informing their pharmacological profiles and potential therapeutic benefits. Well-designed clinical trials are vital to translate laboratory findings into clinical applications, rigorously assessing efficacy, safety, long-term effects, optimal dosages, treatment regimens, and potential interactions with existing therapies such as statins. This research will enhance our understanding of the therapeutic potential of natural products, leading to innovative and personalized lipid management strategies and improved cardiovascular health outcomes.

## 6. Conclusions

This review synthesizes current research on natural products and their influence on HMGCR, a key enzyme in cholesterol biosynthesis. Evidence suggests that a diverse range of natural compounds, including flavonoids, phenolic compounds, and traditional herbal formulations, can modulate HMGCR activity through various mechanisms. These mechanisms include direct inhibition of HMGCR, regulation of HMGCR expression via transcription factors like SREBP-2, activation of AMPK signaling, and modulation of other related pathways, ultimately affecting lipid metabolism and cholesterol homeostasis. Network pharmacology and molecular docking studies further support these findings, identifying HMGCR as a frequent target of natural products across various diseases.

The modulation of HMGCR activity by natural products presents a promising avenue for the treatment of dyslipidemia, cardiovascular diseases, and even cancer, as demonstrated by both in vitro and in vivo studies. While statins are the primary drugs used to inhibit HMGCR, natural products offer a complementary approach with potentially fewer side effects and pleiotropic benefits through their interactions with multiple cellular pathways. Synergistic effects observed when combining natural products with statins suggest the possibility of developing combination therapies that could lower statin dosages, thereby reducing adverse effects while maintaining therapeutic efficacy.

Despite the encouraging findings, significant gaps remain in our understanding of the therapeutic potential of natural HMGCR modulators. While this review provides a detailed synthesis of molecular and cellular findings, it is crucial to acknowledge the inherent limitations associated with relying primarily on in vitro and in silico data. These controlled environments often fail to fully recapitulate the complex physiological conditions and intricate interactions present within a living organism. Consequently, conclusions drawn from such studies, particularly regarding potential clinical translatability, should be interpreted with caution. Further in vivo validation is essential to confirm the observed effects and assess the therapeutic efficacy and safety of the investigated compounds or mechanisms in a more biologically relevant context. More robust in vivo studies and well-designed clinical trials are needed to validate their efficacy, safety, and optimal usage in humans. Future research should focus on elucidating the precise mechanisms of action, optimizing dosage and formulation, and investigating potential drug interactions. Addressing these research gaps is essential to translate the promise of natural products into effective and personalized strategies for managing HMGCR-related conditions and improving overall health. This review acknowledged these limitations and provided a more nuanced and realistic perspective on the current state of the research.

## Abbreviations

The following abbreviations are used in this manuscript:

ABCA1	ATP-binding cassette transporter A1
ABCG5	ATP-binding cassette transporter G5
ACAT	Acyl CoA–cholesterol acyltransferase
AETC	Aqueous extract of *Taxus chinensis* var. *Mairei*
ALA-PS	α-linolenic acid ester of plant sterols
AMPK	AMP-activated protein kinase
AnTT	Annatto-derived tocotrienol
AOF	*Alpiniae oxyphyllae* Fructus
AS	Atherosclerosis
ATGL	Adipose triglyceride lipase
BEE	Black elderberry extract
BMP-2	Bone morphogenetic protein-2
CCL2	Chemokine (C-C motif) ligand 2
CE	Cepharanthine
CI	Combination index
CPT-1	Carnitine palmitoyltransferase 1
CQA	Chlorogenic acid
CRP	C-Reactive Protein
CSF/CSE	Two cocoa shell ingredients, a flour (CSF) and an aqueous extract (CSE)
CVD	Cardiovascular disease
CYP7A1	Cholesterol 7α-hydroxylase
DI-HET	Hydroethanolic extract from *Dillenia indica* leaf
ERAD	Endoplasmic reticulum-associated degradation
ERK	Extracellular signal regulated kinase
ESR1	Estrogen receptor 1
FAS	Fatty acid synthase
FABP	Fatty-acid-binding proteins
FAT	Fatty-acid translocase
FATP	Fatty acid transport proteins
FDFT1	Farnesyl-Diphosphate Farnesyltransferase 1
FFAR	Free fatty acid receptors
FKBP38	FK506-binding protein 8
FOE	Forest onion extract
GINST	A hydrolyzed ginseng extract
GLP-1	Glucagon-like peptide 1
Gyp L	Gypenoside L
HCC	Hepatocellular carcinoma
HDL	High-density lipoprotein
HepG2	Hepatocellular carcinoma cell line
HFD	High-fat diet
HMG-CoA	3-Hydroxy-3-methylglutaryl-CoA
HMGCR	HMG-CoA reductase
HMGCS1	3-Hydroxy-3-methylglutaryl-CoA synthase 1
HSD11B1	11β-Hydroxysteroid dehydrogenase 1
HSYA	Hydroxysafflor yellow A
HUA	Hyperuricemia
IDI1	Isopentenyl-diphosphate delta-isomerase
IRS	Insulin receptor substrate
LDL	Low-density lipoprotein
LDL-C	Low-density lipoprotein cholesterol
LDLR	LDL receptor
LV	Lovastatin
*LXRα*	Liver X receptor α
MAPK8	Mitogen-activated protein kinase 8
MASLD	Metabolic dysfunction-associated steatosis liver disease
mTOR	Mammalian target of rapamycin
MVA	Mevalonate pathway
NGN	Naringenin
NOS2	Nitric oxide synthase 2
OA	Oleic acid
OS	Overall survival
ORR	Objective response rate
PCSK9	Proprotein convertase subtilisin/kexin type 9
PFS	Progression-free survival
PHE	*Protium heptaphyllum* Gum Resin Extract
PI3K-Akt	PI3K (phosphatidylinositol 3-kinase) and Akt (protein kinase B)
p-LKB-1	Phospho-liver kinase B1
PPAR	Peroxisome proliferator-activated receptors
PTGS2	Prostaglandin G/H synthase 2
QTB	Theabrownin from Qingzhuan tea
RAD	Radix *Angelica dahuricae*
RCT	Randomized controlled trials
ROS	Reactive oxygen species
RYR	Red yeast rice
SCLC	Small cell lung cancer
SFP	*Sargassum fusiforme* polysaccharide
SIRT-1	Sirtuin 1
SJG	Sanhua Jiangzhi Granules
SLXG	Shugan Lidan Xiaoshi Granules
Spid A	Schipenindolene A
SQLE	Squalene epoxidase
SR-B1	Scavenger receptor class B type 1
SREBP-2	Sterol regulatory element-binding protein 2
TC	Total cholesterol
TCM	Traditional Chinese Medicine
TG	Triglyceride
TSG	Tetrahydroxy stilbene glucoside
UME	*Ulmus macrocarpa* Hance

## References

[1] Reuter S., Gupta S.C., Chaturvedi M.M., Aggarwal B.B. (2010). Oxidative stress, inflammation, and cancer: How are they linked?. Free Radic. Biol. Med..

[2] Neshat S., Rezaei A., Farid A., Sarallah R., Javanshir S., Ahmadian S., Chatrnour G., Daneii P., Heshmat-Ghahdarijani K. (2022). The tangled web of dyslipidemia and cancer: Is there any association?. J. Res. Med. Sci..

[3] Vekic J., Stromsnes K., Mazzalai S., Zeljkovic A., Rizzo M., Gambini J. (2023). Oxidative Stress, Atherogenic Dyslipidemia, and Cardiovascular Risk. Biomedicines.

[4] Goldstein J.L., Brown M.S. (1990). Regulation of the mevalonate pathway. Nature.

[5] Endo A. (1992). The discovery and development of HMG-CoA reductase inhibitors. J. Lipid Res..

[6] Istvan E.S., Deisenhofer J. (2001). Structural mechanism for statin inhibition of HMG-CoA reductase. Science.

[7] Sever P.S., Dahlöf B., Poulter N.R., Wedel H., Beevers G., Caulfield M., Collins R., Kjeldsen S.E., Kristinsson A., McInnes G.T. (2003). Prevention of coronary and stroke events with atorvastatin in hypertensive patients who have average or lower-than-average cholesterol concentrations, in the Anglo-Scandinavian Cardiac Outcomes Trial--Lipid Lowering Arm (ASCOT-LLA): A multicentre randomised controlled trial. Lancet.

[8] Sever P.S., Poulter N.R., Dahlof B., Wedel H., Beevers G., Caulfield M., Collins R., Kjeldsen S.E., Kristinsson A., McInnes G. (2008). The Anglo-Scandinavian Cardiac Outcomes Trial lipid lowering arm: Extended observations 2 years after trial closure. Eur. Heart J..

[9] Liao J.K., Laufs U. (2005). Pleiotropic effects of statins. Annu. Rev. Pharmacol. Toxicol..

[10] Gomez Sandoval Y.H., Braganza M.V., Daskalopoulou S.S. (2011). Statin discontinuation in high-risk patients: A systematic review of the evidence. Curr. Pharm. Des..

[11] Chaudhuri A. (2020). Frontiers in Lipid Lowering Therapy: To Statins and Beyond. Eur. J. Vasc. Endovasc. Surg..

[12] Mulder K.C., Mulinari F., Franco O.L., Soares M.S., Magalhães B.S., Parachin N.S. (2015). Lovastatin production: From molecular basis to industrial process optimization. Biotechnol. Adv..

[13] Alberts A.W., Chen J., Kuron G., Hunt V., Huff J., Hoffman C., Rothrock J., Lopez M., Joshua H., Harris E. (1980). Mevinolin: A highly potent competitive inhibitor of hydroxymethylglutaryl-coenzyme A reductase and a cholesterol-lowering agent. Proc. Natl. Acad. Sci. USA.

[14] Adel Mehraban M.S., Tabatabaei-Malazy O., Rahimi R., Daniali M., Khashayar P., Larijani B. (2021). Targeting dyslipidemia by herbal medicines: A systematic review of meta-analyses. J. Ethnopharmacol..

[15] Hanis N., Ismail N.A., Ali E.Z. (2025). Systematic review on effectiveness of flavonoids against hypercholesterolemia: Insights from in-silico, in-vitro, and in-vivo studies. Food Chem. Adv..

[16] Cheung B., Sikand G., Dineen E.H., Malik S., Barseghian El-Farra A. (2023). Lipid-Lowering Nutraceuticals for an Integrative Approach to Dyslipidemia. J. Clin. Med..

[17] Asma S.T., Acaroz U., Imre K., Morar A., Shah S.R.A., Hussain S.Z., Arslan-Acaroz D., Demirbas H., Hajrulai-Musliu Z., Istanbullugil F.R. (2022). Natural Products/Bioactive Compounds as a Source of Anticancer Drugs. Cancers.

[18] Zhao L.S., Liu R.F., Kang Y.T., Chen Y.Y., Xiao Y.C., Zhang L.L., Cheng X.R. (2025). Effects of Four Main Active Flavonoids of Coreopsis tinctoria Nutt. on Oleic Acid-Induced Lipid Metabolism and Oxidative Stress in HepG2 Cells. Discov. Med..

[19] Permatasari H.K., Abshori N.F., Syahputra R.A., Harahap U., Amalia N., Kumalawati D.A., Mayulu N., Taslim N.A., Tallei T.E., Tjandrawinata R.R. (2024). Novel Functional Food Properties of Forest Onion (Eleutherine bulbosa Merr.) Phytochemicals for Treating Metabolic Syndrome: New Insights from a Combined Computational and In Vitro Approach. Nutrients.

[20] Wei J., Lv Q., Luan F., Zhang X., Guo D., Zhai B., Chen S., Zou J., Shi Y. (2024). Exploration of potential mechanism of Sanhua Jiangzhi granules for the treatment of hyperlipidemia based on network pharmacology and experimental verification. Fitoterapia.

[21] Han H.J., Song X., Yadav D., Hwang M.S., Lee J.H., Lee C.H., Kim T.H., Lee J.J., Kwon J. (2019). Ulmus macrocarpa Hance modulates lipid metabolism in hyperlipidemia via activation of AMPK pathway. PLoS ONE.

[22] Chen L., Liu Y., Tang Z., Song Z., Cao F., Shi X., Xie P., Wei P., Li M. (2022). Radix Angelica dahuricae extract ameliorates oestrogen deficiency-induced dyslipidaemia in ovariectomized (OVX) rats by modulating the gut microbiota and bile acid signalling. Phytomedicine.

[23] Bhat S., Majeed Y., Yatoo G.N., Hassan S., Khan T., Sofi P.A., Ganai B.A., Fazili K.M., Zargar S.M. (2024). Unravelling effects of phytochemicals from buckwheat on cholesterol metabolism and lipid accumulation in HepG2 cells and its validation through gene expression analysis. Mol. Biol. Rep..

[24] Wang K., Liang C., Cao W., Luo G., Zhong S., Zeng Z., Dai L., Song J.L. (2022). Dietary sinapic acid attenuated high-fat diet-induced lipid metabolism and oxidative stress in male Syrian hamsters. J. Food Biochem..

[25] Lupo M.G., Macchi C., Marchianò S., Cristofani R., Greco M.F., Dall’Acqua S., Chen H., Sirtori C.R., Corsini A., Ruscica M. (2019). Differential effects of red yeast rice, Berberis aristata and Morus alba extracts on PCSK9 and LDL uptake. Nutr. Metab. Cardiovasc. Dis..

[26] Xie P., Guo M., Xie J.B., Xiao M.Y., Qi Y.S., Duan Y., Li F.F., Piao X.L. (2022). Effects of heat-processed Gynostemma pentaphyllum on high-fat diet-fed mice of obesity and functional analysis on network pharmacology and molecular docking strategy. J. Ethnopharmacol..

[27] Lee M.S., Kim J.S., Cho S.M., Lee S.O., Kim S.H., Lee H.J. (2015). Fermented Rhus verniciflua Stokes Extract Exerts an Antihepatic Lipogenic Effect in Oleic-Acid-Induced HepG2 Cells via Upregulation of AMP-Activated Protein Kinase. J. Agric. Food Chem..

[28] Dai S., Zhang G.C., Xiang Y., Liu Y., Wang H., Zhao F., Shu Q. (2024). Taxus chinensis var. mairei (Lemée et Lévl) Cheng et L.K. Fu overcomes the resistance to osimertinib in EGFR-mutant non-small-cell lung cancer via suppression of ERK1/2-related cholesterol biosynthesis. J. Ethnopharmacol..

[29] Braojos C., Rebollo-Hernanz M., Cañas S., Aguilera Y., Gil-Ramírez A., Benítez V., Martín-Cabrejas M.A. (2024). Cocoa shell ingredients improve their lipid-lowering properties under simulated digestion: In vitro and HepG2 cells study. Food Res. Int..

[30] Yang B., Wang W., Jian C., Lv B., He H., Wang M., Li S., Guo Y. (2025). Screening of the Lipid-Lowering Probiotic Lactiplantibacillus Plantarum SDJ09 and its Anti-Obesity Mechanism. Appl. Biochem. Biotechnol..

[31] Zhang S., Yang Y., Zhang R., Gao J., Wu M., Wang J., Sheng J., Sun P. (2024). The Potential Mechanism of Alpiniae oxyphyllae Fructus Against Hyperuricemia: An Integration of Network Pharmacology, Molecular Docking, Molecular Dynamics Simulation, and In Vitro Experiments. Nutrients.

[32] Chen Y., Xie C., Lei Y., Ye D., Wang L., Xiong F., Wu H., He Q., Zhou H., Li L. (2024). Theabrownin from Qingzhuan tea prevents high-fat diet-induced MASLD via regulating intestinal microbiota. Biomed. Pharmacother..

[33] Poornima M.S., Sindhu G., Billu A., Sruthi C.R., Nisha P., Gogoi P., Baishya G., Raghu K.G. (2022). Pretreatment of hydroethanolic extract of Dillenia indica L. attenuates oleic acid induced NAFLD in HepG2 cells via modulating SIRT-1/p-LKB-1/AMPK, HMGCR & PPAR-α signaling pathways. J. Ethnopharmacol..

[34] Mannino G., Iovino P., Lauria A., Genova T., Asteggiano A., Notarbartolo M., Porcu A., Serio G., Chinigò G., Occhipinti A. (2021). Bioactive Triterpenes of Protium heptaphyllum Gum Resin Extract Display Cholesterol-Lowering Potential. Int. J. Mol. Sci..

[35] Zhu Q., Li G., Ma L., Chen B., Zhang D., Gao J., Deng S., Chen Y. (2023). Virgin Camellia Seed Oil Improves Glycolipid Metabolism in the Kidney of High Fat-Fed Rats through AMPK-SREBP Pathway. Nutrients.

[36] Li M., Meng Y., Hong X., Chai H., Huang J., Wang F., Zhang W., Wang J., Liu Q., Xu Y. (2024). Anti-atherosclerotic effect of tetrahydroxy stilbene glucoside via dual-targeting of hepatic lipid metabolisms and aortic M2 macrophage polarization in ApoE(-/-) mice. J. Pharm. Biomed. Anal..

[37] Wang M., Cui B., Gong M., Liu Q., Zhuo X., Lv J., Yang L., Liu X., Wang Z., Dai L. (2022). Arctium lappa leaves based on network pharmacology and experimental validation attenuate atherosclerosis by targeting the AMPK-mediated PPARG/*LXRα* pathway. Biomed. Pharmacother..

[38] Han H., Yan P., Chen L., Luo C., Gao H., Deng Q., Zheng M., Shi Y., Liu L. (2015). Flaxseed Oil Containing α -Linolenic Acid Ester of Plant Sterol Improved Atherosclerosis in ApoE Deficient Mice. Oxid. Med. Cell Longev..

[39] Su X.Z., Zhang L.F., Hu K., An Y., Zhang Q.P., Tang J.W., Yan B.C., Li X.R., Cai J., Li X.N. (2024). Discovery of Natural Potent HMG-CoA Reductase Degraders for Lowering Cholesterol. Angew. Chem. Int. Ed. Engl..

[40] Farrell N., Norris G., Lee S.G., Chun O.K., Blesso C.N. (2015). Anthocyanin-rich black elderberry extract improves markers of HDL function and reduces aortic cholesterol in hyperlipidemic mice. Food Funct..

[41] Chen Y.H., Chiu C.C., Hung S.W., Liu J.Y., Wang Y.C., Lv Q., Hsu C.C., Huang Y.W., Huang W.C., Chuang H.L. (2017). Effects of plant- and animal-based high-fat diets on lipid storage and distribution in environmental bacteria-colonized gnotobiotic mice. Biochem. Biophys. Res. Commun..

[42] Guo C., Zhang L., Zhao M., Ai Y., Liao W., Wan L., Liu Q., Li S., Zeng J., Ma X. (2023). Targeting lipid metabolism with natural products: A novel strategy for gastrointestinal cancer therapy. Phytother. Res..

[43] Zhao F., Ding Z., Chen M., Ji M., Li F. (2024). Cepharanthine as an effective small cell lung cancer inhibitor: Integrated insights from network pharmacology, RNA sequencing, and experimental validation. Front. Pharmacol..

[44] Xiao M.Y., Pei W.J., Li S., Li F.F., Xie P., Luo H.T., Hyun Yoo H., Piao X.L. (2024). Gypenoside L inhibits hepatocellular carcinoma by targeting the SREBP2-HMGCS1 axis and enhancing immune response. Bioorg. Chem..

[45] Metibemu D.S., Akinloye O.A., Akamo A.J., Okoye J.O., Omotuyi I.O. (2021). In-silico HMG-CoA reductase-inhibitory and in-vivo anti-lipidaemic/anticancer effects of carotenoids from Spondias mombin. J. Pharm. Pharmacol..

[46] Hong M.Y., Seeram N.P., Zhang Y., Heber D. (2008). Chinese red yeast rice versus lovastatin effects on prostate cancer cells with and without androgen receptor overexpression. J. Med. Food.

[47] Hong M.Y., Seeram N.P., Zhang Y., Heber D. (2008). Anticancer effects of Chinese red yeast rice versus monacolin K alone on colon cancer cells. J. Nutr. Biochem..

[48] Sangande F., Agustini K., Budipramana K. (2023). Antihyperlipidemic mechanisms of a formula containing Curcuma xanthorrhiza, Sechium edule, and Syzigium polyanthum: In silico and in vitro studies. Comput. Biol. Chem..

[49] Yu H.C., Bai Q.R., Guo J.J., Chen M.Y., Wang L., Tong F.C., Zhang S.L., Wu J. (2024). Elucidating hydroxysafflor yellow A’s multi-target mechanisms against alcoholic liver disease through integrative pharmacology. Phytomedicine.

[50] Wang Y., Wang J., Zhou T., Chen Z., Wang W., Liu B., Li Y. (2024). Investigating the potential mechanism and therapeutic effects of SLXG for cholesterol gallstone treatment. Phytomedicine.

[51] Chen N., Chu Y., Su S., Zhang Q., Zhang L. (2024). Network Pharmacology and Molecular Docking Validation to Explore the Pharmacological Mechanism of Zhuling Decoction against Nephrotic Syndrome. Curr. Pharm. Des..

[52] Sakarwal A., Sen K., Ram H., Chowdhury S., Kashyap P., Shukla S.D., Panwar A. (2025). Neuroprotective Efficacy of Phytoconstituents of Methanolic Shoots Extract of Calligonum polygonoides L. in Hypercholesterolemia-associated Neurodegenerations. Endocr. Metab. Immune Disord. Drug Targets.

[53] Grande F., Occhiuzzi M.A., Perri M.R., Ioele G., Rizzuti B., Statti G., Garofalo A. (2021). Polyphenols from Citrus Tacle(^®^) Extract Endowed with HMGCR Inhibitory Activity: An Antihypercholesterolemia Natural Remedy. Molecules.

[54] Navarro-González I., Pérez-Sánchez H., Martín-Pozuelo G., García-Alonso J., Periago M.J. (2014). The inhibitory effects of bioactive compounds of tomato juice binding to hepatic HMGCR: In vivo study and molecular modelling. PLoS ONE.

[55] Ibrahim A., Shafie N.H., Mohd Esa N., Shafie S.R., Bahari H., Abdullah M.A. (2020). Mikania micrantha Extract Inhibits HMG-CoA Reductase and ACAT2 and Ameliorates Hypercholesterolemia and Lipid Peroxidation in High Cholesterol-Fed Rats. Nutrients.

[56] Xie Z., Li E.W., Gao G., Du Y., Wang M., Wang H., Wang P., Qiao Y., Su Y., Xu J. (2022). Zexie Tang targeting FKBP38/mTOR/SREBPs pathway improves hyperlipidemia. J. Ethnopharmacol..

[57] Ren J., Zuo J., Yin B., Huang D., Wen R., Pei H., Liu J., Zhang Y., Zhu S., Zhen S. (2024). Flaxseed Oil Alleviates PFOS-Induced Liver Injury by Regulating Hepatic Cholesterol Metabolism. J. Agric. Food Chem..

[58] Lee K.H., Jeong E.S., Jang G., Na J.R., Park S., Kang W.S., Kim E., Choi H., Kim J.S., Kim S. (2020). Unripe Rubus coreanus Miquel Extract Containing Ellagic Acid Regulates AMPK, SREBP-2, HMGCR, and INSIG-1 Signaling and Cholesterol Metabolism In Vitro and In Vivo. Nutrients.

[59] Han J.S., Sung J.H., Lee S.K. (2017). Inhibition of Cholesterol Synthesis in HepG2 Cells by GINST-Decreasing HMG-CoA Reductase Expression Via AMP-Activated Protein Kinase. J. Food Sci..

[60] Liu C., Ma J., Sun J., Cheng C., Feng Z., Jiang H., Yang W. (2017). Flavonoid-Rich Extract of Paulownia fortunei Flowers Attenuates Diet-Induced Hyperlipidemia, Hepatic Steatosis and Insulin Resistance in Obesity Mice by AMPK Pathway. Nutrients.

[61] Zhang Y., Si Y., Yao S., Yang N., Song G., Sang H., Zu D., Xu X., Wang J., Qin S. (2013). Celastrus orbiculatus Thunb. decreases athero-susceptibility in lipoproteins and the aorta of guinea pigs fed high fat diet. Lipids.

[62] Lim T., Lee K., Kim R.H., Cha K.H., Koo S.Y., Moon E.C., Hwang K.T. (2022). Black raspberry extract can lower serum LDL cholesterol via modulation of gut microbial composition and serum bile acid profile in rats fed trimethylamine-N-oxide with a high-fat diet. Food Sci. Biotechnol..

[63] Li Z.R., Jia R.B., Wu J., Lin L., Ou Z.R., Liao B., Zhang L., Zhang X., Song G., Zhao M. (2021). Sargassum fusiforme polysaccharide partly replaces acarbose against type 2 diabetes in rats. Int. J. Biol. Macromol..

[64] Wan Hasan W.N., Chin K.Y., Abd Ghafar N., Soelaiman I.N. (2020). Annatto-Derived Tocotrienol Promotes Mineralization of MC3T3-E1 Cells by Enhancing BMP-2 Protein Expression via Inhibiting RhoA Activation and HMG-CoA Reductase Gene Expression. Drug Des. Devel Ther..

[65] Tu L., Sun H., Tang M., Zhao J., Zhang Z., Sun X., He S. (2019). Red raspberry extract (Rubus idaeus L shrub) intake ameliorates hyperlipidemia in HFD-induced mice through PPAR signaling pathway. Food Chem. Toxicol..

[66] Forbes-Hernández T.Y., Giampieri F., Gasparrini M., Afrin S., Mazzoni L., Cordero M.D., Mezzetti B., Quiles J.L., Battino M. (2017). Lipid Accumulation in HepG2 Cells Is Attenuated by Strawberry Extract through AMPK Activation. Nutrients.

[67] Zhang Q., Ye Q., Huang X., Xu A., Liu Y., Qi J., Zhang H., Zhang J. (2020). Revealing active components, action targets and molecular mechanism of Gandi capsule for treating diabetic nephropathy based on network pharmacology strategy. BMC Complement. Med. Ther..

[68] Tallarida R.J. (2001). Drug synergism: Its detection and applications. J. Pharmacol. Exp. Ther..

[69] Chou T.C. (2006). Theoretical basis, experimental design, and computerized simulation of synergism and antagonism in drug combination studies. Pharmacol. Rev..

[70] Greco W.R., Bravo G., Parsons J.C. (1995). The search for synergy: A critical review from a response surface perspective. Pharmacol. Rev..

[71] Hao S., Xiao Y., Lin Y., Mo Z., Chen Y., Peng X., Xiang C., Li Y., Li W. (2016). Chlorogenic acid-enriched extract from Eucommia ulmoides leaves inhibits hepatic lipid accumulation through regulation of cholesterol metabolism in HepG2 cells. Pharm. Biol..

[72] Singh V., Jain M., Misra A., Khanna V., Rana M., Prakash P., Malasoni R., Dwivedi A.K., Dikshit M., Barthwal M.K. (2013). Curcuma oil ameliorates hyperlipidaemia and associated deleterious effects in golden Syrian hamsters. Br. J. Nutr..

[73] Lalu M.M., Montroy J., Begley C.G., Bubela T., Hunniford V., Ripsman D., Wesch N., Kimmelman J., Macleod M., Moher D. (2020). Identifying and understanding factors that affect the translation of therapies from the laboratory to patients: A study protocol. F1000Research.

[74] Contopoulos-Ioannidis D.G., Ntzani E., Ioannidis J.P. (2003). Translation of highly promising basic science research into clinical applications. Am. J. Med..

[75] Saeidnia S., Manayi A., Abdollahi M. (2015). From in vitro Experiments to in vivo and Clinical Studies; Pros and Cons. Curr. Drug Discov. Technol..

[76] Lorian V. (1988). Differences between in vitro and in vivo studies. Antimicrob. Agents Chemother..

[77] Caesar L.K., Cech N.B. (2019). Synergy and antagonism in natural product extracts: When 1 + 1 does not equal 2. Nat. Prod. Rep..

[78] Pezzani R., Salehi B., Vitalini S., Iriti M., Zuñiga F.A., Sharifi-Rad J., Martorell M., Martins N. (2019). Synergistic Effects of Plant Derivatives and Conventional Chemotherapeutic Agents: An Update on the Cancer Perspective. Medicina.

[79] Atanasov A.G., Waltenberger B., Pferschy-Wenzig E.M., Linder T., Wawrosch C., Uhrin P., Temml V., Wang L., Schwaiger S., Heiss E.H. (2015). Discovery and resupply of pharmacologically active plant-derived natural products: A review. Biotechnol. Adv..

[80] Gurib-Fakim A. (2006). Medicinal plants: Traditions of yesterday and drugs of tomorrow. Mol. Aspects Med..

[81] Williamson E.M. (2001). Synergy and other interactions in phytomedicines. Phytomedicine.

[82] Grundy S.M., Stone N.J., Bailey A.L., Beam C., Birtcher K.K., Blumenthal R.S., Braun L.T., de Ferranti S., Faiella-Tommasino J., Forman D.E. (2019). 2018 AHA/ACC/AACVPR/AAPA/ABC/ACPM/ADA/AGS/APhA/ASPC/NLA/PCNA Guideline on the Management of Blood Cholesterol: Executive Summary: A Report of the American College of Cardiology/American Heart Association Task Force on Clinical Practice Guidelines. J. Am. Coll. Cardiol..

[83] Mach F., Baigent C., Catapano A.L., Koskinas K.C., Casula M., Badimon L., Chapman M.J., De Backer G.G., Delgado V., Ference B.A. (2019). 2019 ESC/EAS Guidelines for the management of dyslipidaemias: Lipid modification to reduce cardiovascular risk: The Task Force for the management of dyslipidaemias of the European Society of Cardiology (ESC) and European Atherosclerosis Society (EAS). Eur. Heart J..

[84] Yusuf S., Collins R., Peto R. (1984). Why do we need some large, simple randomized trials?. Statist. Med..

[85] Eisenhauer E.A., Therasse P., Bogaerts J., Schwartz L.H., Sargent D., Ford R., Dancey J., Arbuck S., Gwyther S., Mooney M. (2009). New response evaluation criteria in solid tumours: Revised RECIST guideline (version 1.1). Eur. J. Cancer.

[86] Guedes L., Reis P., Machuqueiro M., Ressaissi A., Pacheco R., Serralheiro M.L. (2019). Bioactivities of Centaurium erythraea (Gentianaceae) Decoctions: Antioxidant Activity, Enzyme Inhibition and Docking Studies. Molecules.

[87] Atanasov A.G., Zotchev S.B., Dirsch V.M., Orhan I.E., Banach M., Rollinger J.M., Barreca D., Weckwerth W., Bauer R., Bayer E.A. (2021). Natural products in drug discovery: Advances and opportunities. Nat. Rev. Drug Discov..

[88] Butler M.S. (2004). The role of natural product chemistry in drug discovery. J. Nat. Prod..

[89] Harvey A.L., Edrada-Ebel R., Quinn R.J. (2015). The re-emergence of natural products for drug discovery in the genomics era. Nat. Rev. Drug Discov..

[90] Miron A., Aprotosoaie A.C., Trifan A., Xiao J. (2017). Flavonoids as modulators of metabolic enzymes and drug transporters. Ann. N. Y. Acad. Sci..

[91] Eggertsen R., Andreasson A., Andrén L. (2007). Effects of treatment with a commercially available St John’s Wort product (Movina) on cholesterol levels in patients with hypercholesterolemia treated with simvastatin. Scand. J. Prim. Health Care.

[92] Lilja J.J., Kivistö K.T., Neuvonen P.J. (1998). Grapefruit juice-simvastatin interaction: Effect on serum concentrations of simvastatin, simvastatin acid, and HMG-CoA reductase inhibitors. Clin. Pharmacol. Ther..

[93] Gurley B.J. (2025). Clinically Relevant Herb–Drug Interactions: A 30-Year Historical Assessment. J. Diet. Suppl..

[94] Klein-Junior L.C., de Souza M.R., Viaene J., Bresolin T.M.B., de Gasper A.L., Henriques A.T., Heyden Y.V. (2021). Quality Control of Herbal Medicines: From Traditional Techniques to State-of-the-art Approaches. Planta Med..

[95] Yin J., Xing H., Ye J. (2008). Efficacy of berberine in patients with type 2 diabetes mellitus. Metabolism.

[96] Halbert S.C., French B., Gordon R.Y., Farrar J.T., Schmitz K., Morris P.B., Thompson P.D., Rader D.J., Becker D.J. (2010). Tolerability of red yeast rice (2,400 mg twice daily) versus pravastatin (20 mg twice daily) in patients with previous statin intolerance. Am. J. Cardiol..

